# Targeted Delivery of Celastrol by GA-Modified Liposomal Calcium Carbonate Nanoparticles to Enhance Antitumor Efficacy Against Breast Cancer

**DOI:** 10.3390/pharmaceutics16111382

**Published:** 2024-10-27

**Authors:** Wei Zhang, Jiping Li, Liling Yue, Chenfeng Ji

**Affiliations:** 1 Engineering Research Center for Medicine, College of Pharmacy, Harbin University of Commerce, Harbin 150028, China; zhv1110@qmu.edu.cn; 2 School of Public Health, Qiqihar Medical University, Qiqihar 161006, China; lijipinglyh@163.com; 3 Office of Academic Research, Qiqihar Medical University, Qiqihar 161006, China; yuell1025@qmu.edu.cn

**Keywords:** celastrol, calcium carbonate, lipid coated, nanoparticles, cancer treatment

## Abstract

Background/Objectives: Breast cancer, a leading health threat affecting millions worldwide, requires effective therapeutic interventions. Celastrol (CEL), despite its antitumor potential, is limited by poor solubility and stability. This study aimed to enhance CEL’s efficacy by encapsulating it within glycyrrhizic acid (GA)-modified lipid calcium carbonate (LCC) nanoparticles for targeted breast cancer therapy. Methods: The 4T1 mouse breast cancer cells were used for the study. GA-LCC-CEL nanoparticles were prepared using a gas diffusion method and a thin-film dispersion method. GA-LCC-CEL were characterized using the zeta-potential, dynamic light scattering and transmission electron microscope (TEM). The in vitro release behavior of nanoparticles was assessed using the in vitro dialysis diffusion method. Cellular uptake was examined using flow cytometry and confocal microscopy. Intracellular ROS and Rhodamine 123 levels were observed under fluorescence microscopy. MTT and colony formation assays assessed cytotoxicity and proliferation, and apoptosis was analyzed by Annexin V-FITC/PI staining. Wound healing and transwell assays evaluated migration, and Western blotting confirmed protein expression changes related to apoptosis and migration. Results: GA-LCC-CEL nanoparticles displayed a well-defined core-shell structure with a uniform size distribution. They showed enhanced anti-proliferative and pro-apoptotic effects against 4T1 cells and significantly reduced breast cancer cell invasion and migration. Additionally, GA-LCC-CEL modulated epithelial-mesenchymal transition (EMT) protein expression, downregulating Snail and ZEB1, and upregulating E-cadherin. Conclusions: GA-LCC-CEL nanoparticles represent a promising targeted drug delivery approach for breast cancer, enhancing CEL’s antitumor efficacy and potentially inhibiting cancer progression by modulating EMT-related proteins.

## 1. Introduction

Breast cancer is a prevalent malignancy that poses a substantial threat to the health and lives of women globally [1]. The progression of breast cancer, particularly triple-negative breast cancer (TNBC), involves the metastasis of malignant epithelial cells from the primary site to distant organs, leading to the formation of secondary tumors [2]. Conventional chemotherapy remains the primary treatment approach for breast cancer. However, traditional chemotherapeutic agents exhibit nonspecific distributions and mechanisms of action, along with adverse reactions and toxic side effects, which can compromise treatment efficacy [3,4].

Celastrol (CEL), also known as tripterine, is an active compound derived from the roots of Tripterygium wilfordii Hook. f. (TWFH) or related Tripterygium species. Recent studies have demonstrated that CEL exhibits a broad spectrum of pharmacological activities, including potent anti-inflammatory, antitumor, antioxidant, immunosuppressive, and neuroprotective effects [5,6,7,8,9]. In 2007, CEL was identified as one of the most promising candidates for development into a natural pharmaceutical [10]. CEL exhibits remarkable efficacy in treating various diseases and interacts with numerous cellular targets. Moreover, its exceptional antitumor potency in various tumor cells and animal models has garnered significant interest among cancer researchers [11]. Recent studies have revealed that CEL exerts its antitumor effects through various mechanisms, including the PI3K/Akt/mTOR pathway [12], mitochondrial dysfunction [13], ROS accumulation [14], the induction of apoptosis and cell cycle arrest [15], and the modulation of NF-κB signaling [16]. However, the poor water solubility, low bioavailability, and significant toxicity of CEL severely limit its clinical application. Therefore, it is imperative to develop novel strategies to enhance the aqueous solubility, reduce the toxicity, and increase the bioavailability of CEL.

The limitations of CEL have led to the development of various formulation strategies aimed at enhancing its therapeutic efficacy in vivo by improving the bioavailability and solubility and reducing the toxicity. The challenges associated with CEL toxicity have been extensively investigated, and nanotechnology-based approaches have been widely explored to address them. CEL delivery to the tumor site has been achieved using nanoparticles with active or passive targeting while simultaneously controlling the release mechanisms. Among these approaches, calcium carbonate nanocarriers (CCNPs) stand out due to their chemical similarity with human hard tissues (bones, teeth) and intracellular signaling pathway ions and their ability to prevent damage to normal tissues, thereby distinguishing them from other inorganic material nanocarriers [17]. The remarkable characteristics of high porosity, excellent pH responsiveness, biocompatibility, biodegradability, in vivo stability, and calcium ion (Ca^2+^) regulation have generated significant research interest in calcium carbonate nanoparticles [18,19]. However, their tendency for aggregation and instability significantly hinders clinical applications when exposed to aqueous solutions [20,21].

Glycyrrhiza radix (GLR), a Chinese herbal medicine, is well-known for its wide spectrum of biological activities. It primarily functions to alleviate heat, to detoxify, and to enhance the compatibility of various medications. When used in conjunction with toxic traditional Chinese medicines, licorice effectively reduces bodily harm and adverse reactions [22]. For example, GLR, when combined with TWFH, shows an ameliorative effect on acute and chronic liver injuries induced by TWFH. GA, a key component of GLR, has been scientifically demonstrated to possess hepatoprotective, anti-inflammatory, antioxidant, and antitumor properties [23]. Additionally, GA has significant potential in reducing the hepatotoxicity, nephrotoxicity, and neurotoxicity caused by chemotherapy drugs [24,25]. Thus, given GA’s exceptional physical, chemical, and biological properties, using GA instead of cholesterol for liposome modification not only addresses the limitations of cholesterol in conventional liposomes but also improves liposomal physiological stability. The aforementioned effects suggest that GA may be an ideal carrier material for first-line anticancer drugs.

**Scheme 1:**
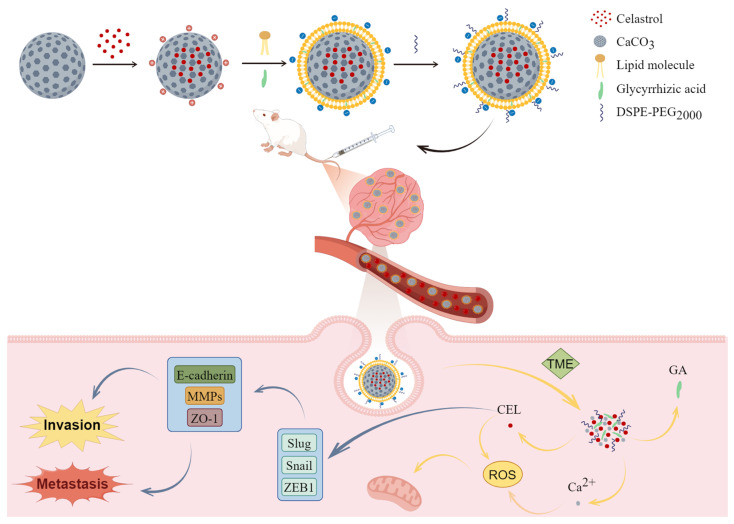
Graphic illustration of the preparation strategy and the antitumor function of GA-LCC-CEL nanoparticles.

In this study, calcium carbonate nanoparticles were formulated and loaded with cel (CCNP-CEL). GA was selected as an alternative to cholesterol when in combination with a phospholipid bilayer characterized by strong cellular affinity and biocompatibility. By leveraging the positive charges of CCNP, we developed a core–shell loading system with lipid nanocarriers coated onto CCNP through electrostatic interactions (GA-LCC-CEL) (Scheme 1, illustrated using Figdraw). This study includes the synthesis, characterization, and in vitro assessment of GA-LCC-CEL nanoparticles for inhibiting breast cancer growth and metastasis. This investigation of GA-LCC-CEL is expected to offer innovative insights into the formulation of toxic natural compounds and to introduce a novel approach to targeted breast cancer therapy.

## 2. Materials and Methods

### 2.1. Materials

CEL (>98%) and GA (>98%) were obtained from Macklin Biotechnology Co., Ltd. (Shanghai, China). Calcium chloride dihydrate (CaCl_2_·2H_2_O) and ammonium bicarbonate (NH_4_HCO_3_) were purchased from Sinopharm Chemical Reagent Co., Ltd. (Shanghai, China). DSPE-PEG2000 was purchased from ToYong Biotech Co., Ltd. (Shanghai, China). Antibodies against Bcl-2 and Bax were obtained from Abcam (Cambridge, UK), while antibodies against β-actin, Snail, Slug, ZEB1, and E-cadherin were obtained from Cell Signaling Technology (Danvers, MA, USA). Antibodies against ZO-1, MMP2, and MMP9 were obtained from Proteintech Group, Inc. (Chicago, IL, USA), while rabbit anti-mouse IgG and goat anti-rabbit IgG were obtained from Cell Signaling Technology (Danvers, USA). Fetal bovine serum (FBS), RPMI-1640 medium, and L-15 were obtained from Gibco BRL (Shanghai, China). The Annexin V-FITC/PI apoptosis detection kit was purchased from ComWin Biotech Co., Ltd. (Beijing, China). 3-[4,5-Dimethylthiazol-2-yl]-2,5-diphenyltetrazolium bromide (MTT) was supplied by Beyotime Biotechnology Co., Ltd. (Shanghai, China).

### 2.2. Cell Culture

4T1 mouse breast cancer cells (RRID: CVCL_0125) were obtained from the Institute of Basic Medical Sciences, Chinese Academy of Medical Sciences (Beijing, China). 4T1 cells were cultured in RPMI-1640 medium supplemented with 10% fetal bovine serum, 100 IU/mL penicillin, and 100 μg/mL streptomycin in a 5% CO_2_ and 37 °C incubator.

### 2.3. Preparation and Characterization of CEL-Loaded GA/LCC Core-Shell Nanoparticles

The nanoparticles were prepared using a gas diffusion method and a thin-film dispersion method, as described previously [26]. Briefly, 5 mg of CEL and 150 mg of CaCl_2_·2H_2_O were dissolved in 100 mL of absolute ethanol, and the container was covered with tinfoil. The beaker was then placed in a vacuum dryer with an appropriate amount of dried NH_4_HCO_3_. The reaction system was allowed to proceed for 24 h at 26 °C in a vacuum environment. After the reaction, the suspension was centrifuged at 12,000× *g* for 10 min, and the CCNP-CEL nanoparticles were dispersed in absolute ethanol and sonicated using a probe ultrasonic instrument (Branson, MO, USA) for further use. Drug-free CCNPs were prepared using the same procedure, without CEL.

The CCNP-CEL nanoparticles were first dispersed in a mixed solution of ethanol and chloroform (3:1, *v*/*v*) containing PL and GA (PL = 30:6, *w*/*w*) and then stirred at 37 °C for 24 h. The mixture was then centrifuged at 3000× *g* for 10 min to remove unbound PL. DSPE-PEG2000 (PL: DSPE-PEG2000 = 30:6, *w*/*w*) was added to the supernatant, and the mixture was stirred for an additional 30 min. The mixed solution was then transferred to a round-bottom flask and subjected to rotary evaporation at 37 °C to form a film using a rotary evaporator (RE100-Pro, Beijing DLAB Scientific Co., Ltd., Beijing, China). The film was dissolved in PBS to produce a GA-LCC-CEL emulsion. A schematic illustration of GA-LCC-CEL synthesis was created using Figdraw. The preparation procedure for nanoparticles loaded with Cou6 or DiR followed the same method, with CEL being replaced by Cou6 or DiR.

The structures and morphologies of the nanoparticles were analyzed using transmission electron microscopy (TEM; JEOL JEM-1200EX, Japan). A 10 μL diluted sample of the nanoparticles was placed on a copper grid and subjected to negative staining with 2% phosphotungstic acid solution. After air-drying at room temperature, the samples were observed using the TEM instrument (TEM; JEOL JEM-1200EX, Tokyo, Japan) operating at 100 kV. The mean particle size, polydispersity index (PDI), and zeta potential values of the nanoparticles were determined using dynamic light scattering (DLS) with the Nicomp 380ZLS particle size determination system (PSS; New York, NY, USA). The encapsulation efficiency (EE) of CEL in each nanoparticle was analyzed using an HPLC system.

To assess the stabilities of the nanoparticles in serum during systemic circulation, a stability assay was conducted in PBS supplemented with 20% FBS by monitoring changes in the mean size and PDI values. GA-LCC-CEL nanoparticles were suspended in PBS containing FBS and agitated at 150 rpm and 37 °C. At specific time intervals, samples were collected for particle size and PDI measurements.

### 2.4. In Vitro Drug Release Assay

The in vitro release behaviors of free CCNP-CEL and GA-LCC-CEL were assessed using the in vitro dialysis diffusion method. For the release studies, 2.0 mL of CCNP-CEL and GA-LCC-CEL solutions were each enclosed within dialysis bags (MWCO12−14 kDa). Then, the dialysis bags were immersed in pH-adjusted PBS (pH 7.4, pH 6.5, and pH 5.5) containing 0.2% Tween 80 (*w*/*w*) solutions and agitated at 37 °C for a duration of 24 h at a speed of 100 rpm. At predetermined time intervals, samples of the release medium (1.0 mL) were collected and replaced with an equal volume of fresh release medium. The concentration of CEL released in vitro was determined by high-performance liquid chromatography (HPLC).

### 2.5. In Vitro Cellular Uptake

To investigate the in vitro localization and uptake of GA-LCC-CEL, 4T1 cells were seeded at a density of 1 × 10^5^ cells per well in six-well plates. After incubation at 37 °C for 24 h, the cells were treated with an equivalent concentration of Cou6. Subsequently, serum-free medium containing CCNPs-Cou6, LCC-Cou6, and GA-LCC-Cou6 was added and incubated for 1 h. The cells were then collected, washed three times with chilled PBS, detached by trypsinization, centrifuged, and transferred to flow cytometry tubes. The fluorescence intensities of the cells were measured using a FACScan flow cytometer (BD FACSCalibur, San Jose, CA, USA) with an excitation wavelength of 488 nm and a detection wavelength of 560 nm.

For confocal microscopy analysis, after treatment with various Cou6 formulations for 1 h at 37 °C, the cells were washed three times with pre-cooled PBS and fixed in 4% paraformaldehyde for 20 min. The nuclei were then stained with Hoechst 33342 for 20 min. Finally, the fluorescence signals of the 4T1 cells were observed and analyzed using an LSM710 laser confocal microscope (Zeiss, Jena, Germany).

### 2.6. Determination of Intracellular ROS Concentration

4T1 cells in the logarithmic growth phase were suspended and seeded in a six-well cell culture plate, followed by overnight incubation. After removing the original culture medium, fresh medium containing free CEL, CCNP-CEL, and GA-LCC-CEL was added to the cells for a 12-h exposure. Serum-free medium was supplemented with 10 μM DCFH-DA, followed by a 30-min incubation and three washes with PBS. The cells were then treated with 10 μg/mL Hoechst 33342 and incubated at 37 °C for 10 min. After three additional washes with PBS, intracellular ROS levels were observed using an inverted fluorescence microscope.

### 2.7. Rhodamine 123 Staining

4T1 cells were inoculated at a density of 1 × 10^6^ cells/mL in a six-well plate and cultured overnight. The medium was replaced with CEL-, LCC-CEL-, and GA-LCC-CEL-containing medium for a 12-h exposure period. Subsequently, the cells were treated with Rhodamine 123 solution (10 μg/mL) and incubated in a 5% CO_2_ incubator at 37 °C for 10 min. Afterward, the cells were washed three times with PBS. Next, Hoechst 33342 solution (10 μg/mL) was added to the cells and incubated at 37 °C for another 10 min. Following this process, the cells were washed three times with PBS before being observed and photographed under an inverted fluorescence microscope with an excitation wavelength of 505 nm and a detection wavelength of 534 nm.

### 2.8. In Vitro Cytotoxicity and Apoptosis Assay

In this experiment, the MTT assay was used to assess cytotoxicity by measuring cell viability. A suspension of 4T1 cells in the logarithmic growth phase was prepared and added to a 96-well plate at a concentration of 1 × 10^5^ cells/mL. The suspension was then incubated in a constant-temperature incubator at 37 °C for 24 h, followed by the removal of the culture medium. Fresh medium containing free CEL, LCC-CEL, and GA-LCC-CEL (with final CEL concentrations ranging from 0.125 to 32 μM) was added precisely. Untreated wells served as controls. After an additional 24-h incubation period, 20 μL of MTT solution (5 mg/mL) was added to each well for another 4 h. After discarding the supernatant, 150 μL of DMSO was added per well, and the crystals were dissolved by shaking on a constant-temperature shaker for 10 min. Absorbance at a wavelength of 490 nm was measured using a plate reader (Tecan Safire2, Männedorf, Switzerland).

To further assess the proliferative capacities of the cells, a cell colony formation assay was used. Cells in the logarithmic growth phase were trypsinized, seeded at a density of 1000 cells per well in a six-well plate, and incubated at 37 °C for 24 h. The medium was then replaced with CEL-, LCC-CEL-, and GA-LCC-CEL-containing medium for an additional 14 days. After colony formation, the cells were washed once with PBS, fixed with 4% paraformaldehyde for 30 min, rinsed with PBS, stained with crystal violet solution (1 mL per well) for 15 min, and washed three times with PBS. Finally, the cells were air-dried and photographed.

Cell apoptosis was analyzed using Annexin V-FITC/PI staining and flow cytometry. 4T1 cells were cultured in 35-mm cell culture dishes and treated with CEL, LCC-CEL, and GA-LCC-CEL for 24 h. The harvested cells were washed twice with PBS and then incubated with Annexin V-FITC and PI for 15 min in the dark. After incubation, the stained cells were quantified and analyzed using a FACScan flow cytometer. The flow cytometry parameters were as follows. A 488 nm laser was used to excite FITC, detecting green fluorescence of Annexin V-FITC at an emission wavelength of 525 nm. A 561-nm laser was used to excite PI, detecting its red fluorescence at an emission wavelength of 640 nm.

### 2.9. In Vitro Cell Migration and Invasion Tests

For the scratch wound healing assay, 4T1 cells were seeded in six-well plates, and straight wounds were gently created using a 20 μL pipette tip after 24 h of incubation. The cells were then washed with PBS to remove any floating cells. The cells were then incubated with CEL, LCC-CEL, and GA-LCC-CEL. Cell migration was monitored over 48 h using an inverted microscope (Zeiss, Axio Observer A1).

For transwell invasion assays, transwell chambers were pre-coated with 50 μL of Matrigel (Corning, Somerville, MA, USA). 4T1 cells were incubated with various CEL formulations for 24 h. The cells were trypsinized and suspended in serum-free RPMI-1640 medium. A 200 μL cell suspension containing 1 × 10^5^ cells was seeded into the upper chamber, and 600 μL of complete RPMI-1640 medium was added to the lower chamber and incubated for another 24 h. Non-invasive cells in the upper chamber were removed using a cotton swab. Cells attached to the bottom of the membrane were fixed with 4% paraformaldehyde, stained with 0.5% crystal violet for 15 min, and observed under an inverted microscope (Zeiss, Axio Observer A1).

### 2.10. Inhibitory Effect on the Tumor Spheroids

4T1 cells in the logarithmic growth phase were digested, resuspended, and seeded at a density of 1000 cells per well into 96-well plates pre-coated with 2% agarose. The plates were incubated at 37 °C until tumor spheroids measuring 300–400 μm in diameter were formed. The tumor spheroids were then exposed to free CEL, LCC-CEL, and GA-LCC-CEL at equimolar concentrations of CEL. Growth inhibition was assessed using an inverted microscope (Zeiss, Axio Observer A1).

### 2.11. In Vitro Western Blotting Studies

The expression levels of apoptosis- and migration-related proteins were assessed using Western blot analysis. 4T1 cells were treated with free CEL, LCC-CEL, and GA-LCC-CEL for 24 h. The cells were then harvested and lysed, and the protein contents in the lysates were quantified using the BCA Protein Assay Kit (Cwbio, Taizhou, China). The proteins were separated by 8%, 10%, and 12% SDS-PAGE and transferred onto nitrocellulose membranes.

The membranes were incubated with 5% non-fat dry milk for 2 h to block non-specific binding. The membranes were then probed with specific primary antibodies against Bcl-2 (1:1000 dilution, ab182858; Abcam, Cambridge, UK), Bax (1:1500 dilution, ab182733; Abcam), β-actin (1:2000, #4970, CST, Hong Kong, China), Snail (1:500, #3879, CST), Slug (1:800, #9585, CST), ZEB1 (1:500, #70512, CST), E-cadherin (1:500, #3195, CST), ZO-1 (1:500, 21773-1-AP, Proteintech Group, Rosemont, IL, USA), and MMP2 and MMP9 (1:500 dilution, 10373-2-AP, 10375-2-AP; Proteintech Group) at 4 °C for overnight binding. The membranes were washed three times with TBST and incubated for 90 min with rabbit anti-mouse IgG and goat anti-rabbit IgG antibodies (1:2000, #7076, #7074, CST). Protein bands were visualized using enhanced chemiluminescence (ECL) detection with the ChemiDoc™ MP imaging system (Bio-Rad, Hercules, CA, USA).

### 2.12. Statistical Analysis

All data were analyzed using IBM SPSS Statistics software, version 27. Statistical significance was performed using Student’s *t*-test, one-way ANOVA, and the Dunnett-*t* test. Data were presented as the mean ± standard deviation (SD) of at least three independent experiments. The differences were considered statistically significant when *p* < 0.05.

## 3. Results and Discussion

### 3.1. Preparation and Characterization of CEL-Loaded GA/LCC Core-Shell Nanoparticles

**Figure 1:**
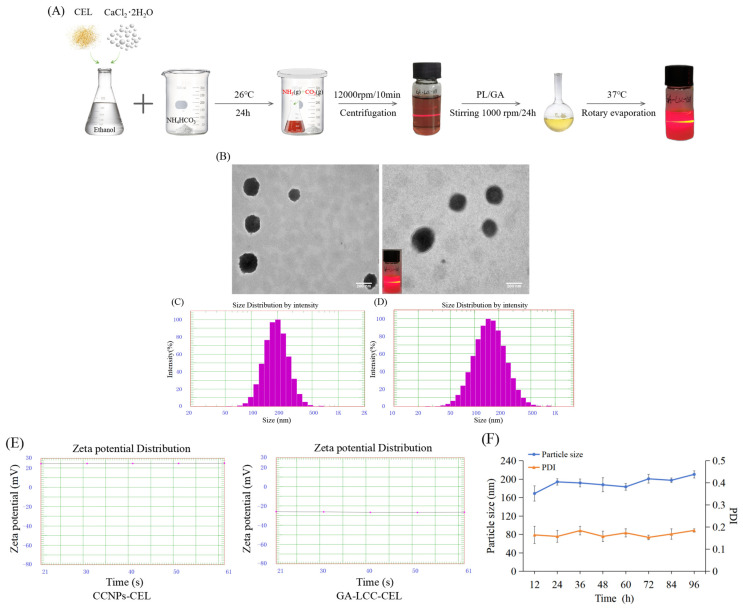
Synthesis and characterization of GA-LCC-CEL. (**A**) Schematic illustration of GA-LCC-CEL synthesis. (**B**) Representative TEM images and photographs of CCNP-CEL and GA-LCC-CEL. (**C**) Hydrodynamic size of GA-LCC-CEL. (**D**) Hydrodynamic size of CCNP-CEL. (**E**) Zeta potential of CCNP-CEL and GA-LCC-CEL. (**F**) Stability studies of GA-LCC-CEL within 4 days.

CCNPs could be synthesized through microemulsion, chemical precipitation, and gas diffusion reactions [27,28,29]. The CCNPs were synthesized in accordance with a previous report with some modifications [30]. CCNPs could be easily prepared using ethanol as the reaction medium and NH_4_HCO_3_ as the source of CO_2_. The continuous decomposition of NH_4_HCO_3_ resulted in the formation of NH_3_ and CO_2_. Subsequently, the CO_2_ dissolved in ethanol and reacted with Ca^2^⁺ from CaCl_2_·2H_2_O to form CCNPs. CEL was immersed in a CaCl_2_·2H_2_O ethanol solution and encapsulated within CCNPs to synthesize CCNP-CEL particles at a designated temperature. The CCNP-CEL particles were then modified with PL and GA, which was followed by surface modification with polyethylene glycol (PEG) via solvent diffusion to obtain CEL-loaded composite lipid nanoparticles (GA-LCC-CEL). The simplified synthesis procedure is illustrated in Figure 1A.

The morphological observation of CCNP-CEL and GA-LCC-CEL was conducted using TEM. As shown in Figure 1B, both CCNP-CEL and GA-LCC-CEL displayed good distributions on the copper grid with distinct boundaries, forming spherical nanoparticles characterized by their size distributions. Dynamic light scattering (DLS) analysis revealed that GA-LCC-CEL nanoparticles had a mean diameter of approximately 206 ± 19 nm (Figure 1C). After modification with the lipid bilayer and DSPE-PEG, both TEM and DLS analysis showed a slight increase in size compared to bare CCNP-CEL (162± 23 nm) (Figure 1D) nanoparticles, indicating the presence of a core–shell structure. Furthermore, a light-colored outer shell surrounded the dark CCNP-CEL core, likely due to the different electron penetration capabilities of CCNP-CEL and the lipid bilayer. Additionally, the nanoparticles in both solutions were transparent and exhibited clearly observable Tyndall effects when an incident beam passed through, indicating their stability as colloidal systems.

Previous results have demonstrated that the zeta potential of calcium carbonate particles is strongly related to the concentration of Ca^2^⁺ during the synthesis process. Therefore, additional Ca^2^⁺ is adsorbed onto the surface of CCNP-CEL nanoparticles, resulting in a positive zeta potential. A zeta sizer was used to investigate the surface potential of CCNP-CEL, revealing an average positive zeta potential of +25.37 ± 2.3 mV for CCNP-CEL (Figure 1E), consistent with previous findings [31]. Subsequent surface modifications with GA, PL, and DSPE-PEG resulted in a sharp reversal of the surface potential to −27.96 ± 2.5 mV for GA-LCC-CEL. This suggests that CCNP-CEL were successfully coated by the liposomal surface, as indicated by the size increase and charge reversal from positive to negative potential observed in GA-LCC-CEL. Additionally, the time-dependent size and polydispersity index (PDI) of GA-LCC-CEL in PBS containing 20% fetal bovine serum, as well as a 4-day storage stability test, showed negligible changes, indicating good stability in a blood circulation-mimicking environment (Figure 1F).

### 3.2. In Vitro Drug Release Assay

**Figure 2:**
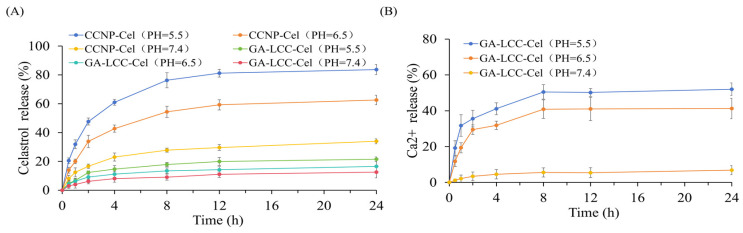
Extracellular release properties of GA-LCC-CEL. (**A**) Time-dependent CEL release profile of GA-LCC-CEL at different pH values. (**B**) Time-dependent Ca^2+^ release profile of GA-LCC-CEL at different pH values. Data are shown as mean ± SD (*n* = 3).

It is widely acknowledged that breast tumors exhibit an acidic microenvironment, with pH values ranging from 6.8 in the periphery and 5.0 in endosomes/lysosomes of cancer cells to 7.4 in blood circulation [32,33]. To investigate the pH-sensitive properties of GA-LCC-CEL nanoparticles, we conducted a release experiment using phosphate buffer saline (PBS) at different pH values through the classical dialysis method over time intervals up to 24 h. The in vitro drug release profile of GA-LCC-CEL under various physiological conditions was thoroughly examined. The cumulative release profile of CEL from liposomes is depicted in Figure 2A. The graph indicates that both in pH 5.0 and 6.8 PBS, CCNP-CEL exhibited relatively fast CEL release during the initial phase. This rapid release could be attributed to the ease of dissolution or phase transition, as supported by previous studies [29]. Furthermore, the cumulative release of CCNP-CEL increased as the pH value decreased, with a higher drug release observed in acidic solutions (pH 5.0) compared to neutral buffers (pH 7.4). These findings suggest that CCNP-CEL exhibits pH sensitivity towards calcium carbonate. Notably, the cumulative release of CEL from GA-LCC-CEL was significantly slower compared to CCNP-CEL under identical conditions. This delay is likely due to GA modification, which retarded CEL release from the core-shell nanoparticles. For example, GA-LCC-CEL demonstrated slower release rates (21.56% at pH 5.0, 16.57% at pH 6.8, and 12.52% at pH 7.4) compared to CCNP-CEL over 24 h. This indicates that the proposed drug delivery system provides a substantial protective effect on CCNPs, effectively reducing rapid drug leakage during in vivo circulation. This result may be attributed to the GA molecules modifying the surface of the liposomes, which potentially decreases fluidity and membrane permeability of the lipid bilayer, thereby enhancing bilayer stability and organization [24,34]. Additionally, the dense hydrophilic GA coating surrounding the liposomes acts as a barrier, restricting CEL dispersion from GA-LCC-CEL and controlling its release into the medium.

Moreover, we assessed the Ca^2^⁺ release behavior of GA-LCC-CEL in PBS at different pH values. As shown in Figure 2B, GA-LCC-CEL maintained a relatively stable state in a pH 7.4 buffer solution, while noticeable Ca^2^⁺ release was observed under acidic conditions (pH 5.0 and pH 6.8). GA-LCC-CEL demonstrated faster and more substantial Ca^2^⁺ release in the pH 5.0 environment compared to pH 6.8. Thus, GA-LCC-CEL effectively responds to acidic environments without degradation in neutral settings, potentially exerting an antitumor effect by inducing Ca^2^⁺ overload within tumors and triggering Ca^2^⁺-mediated mitochondrial dysfunction.

### 3.3. In Vitro Cellular Uptake

**Figure 3:**
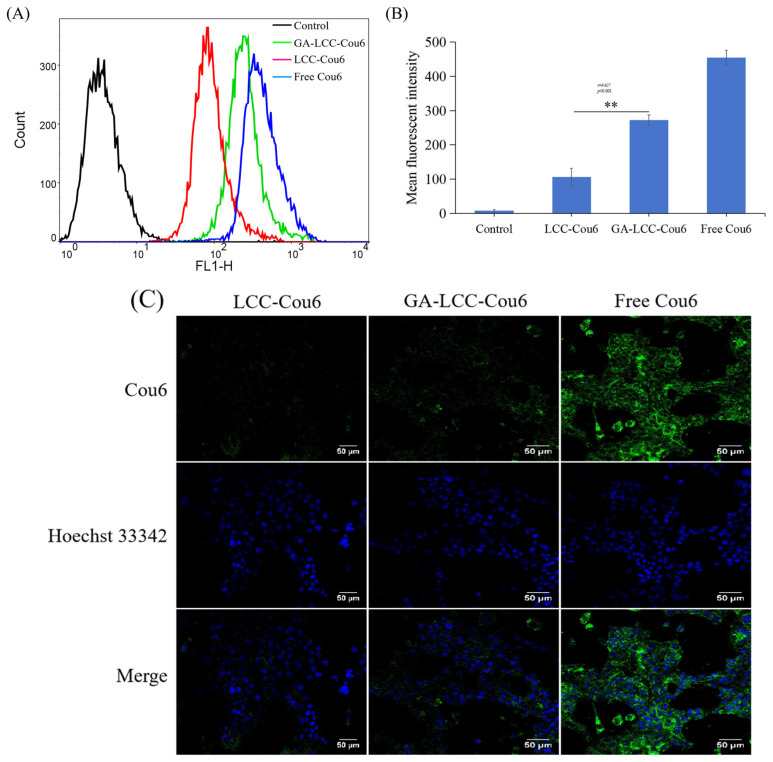
Intracellular uptake study of free Cou 6, LCC-Cou6 and GA-LCC-Cou6 on 4T1 cells after incubation for 2 h at 37 °C. (**A**) Flow cytometric quantitative determination of Cou 6 uptake. (**B**) Quantitative analysis of Cou6 uptake based on flow cytometric plots. The results are shown as a mean fluorescence intensity ± SD (*n* = 3), ** *p* < 0.01. (**C**) Laser scanning confocal microscopy images showing the intracellular distribution of nanoparticles in 4T1 cells. Green and blue indicate the fluorescence of Cou6 and Hoechst 33342, respectively.

To investigate the role of GA in enhancing cellular uptake, we initially examined uptake using flow cytometry. In this study, both LCC and GA-LCC nanoparticles were labeled with the Cou6 green fluorescence probe. As depicted in Figure 3A,B, the mean fluorescence intensity was ranked in the following order: control < LCC-Cou6 < GA-LCC-Cou6 < free Cou6. The cellular uptake of GA-LCC-Cou6 was found to be higher than that of LCC-Cou6. The difference observed between LCC-Cou6 and GA-LCC-Cou6 could be attributed to the GA modification, as their compositions were otherwise similar. The primary uptake mechanism for GA-LCC-Cou6 was likely GA-mediated endocytosis. According to reports, GA could increase the permeabilities of erythrocyte membranes by penetrating the lipid bilayer. Its self-aggregation in water facilitated adherence to the membrane surface and subsequent insertion into a suitable cavity within the bilayer [35,36]. The opening of tight junctions, penetration of water molecules into the membrane, and pore formation by GL may facilitate passive transport across the membrane for both water-soluble polar and non-polar molecules [37].

To gain further insight into cellular uptake, we utilized confocal laser scanning microscopy (CLSM) to observe Cou6-loaded nanoparticles (Figure 3C). The results were consistent with those from flow cytometry. A stronger fluorescence signal (green) was observed in 4T1 cells incubated with GA-LCC-Cou6, indicating a significantly higher uptake of Cou6 compared to the LCC-Cou6 group. Blue represents the nuclear dye Hoechst 33342, which was used for nuclear localization. Consequently, GA-based drug delivery systems had the potential to enhance both in vitro solubility and in vivo cellular penetration of core drugs.

### 3.4. In Vitro Mitochondrial Dysfunction

Early-stage analysis of celastrol-interacting proteins using the human proteome chip revealed that CEL could enhance ROS accumulation by binding to and inhibiting the activities of STAT3, PRDX3, and other proteins [38,39]. This led to ROS accumulation, the disruption of mitochondrial membrane potential, the upregulation of oxidative stress levels, and the subsequent induction of cell death [40,41]. In addition, mitochondria, which are responsible for sensing and regulating intracellular Ca^2^⁺ concentrations, sequester excessive Ca^2^⁺ when cytoplasmic Ca^2^⁺ levels become too high [42,43,44]. Current research suggests that mitochondrial Ca^2^⁺ overload could likely induce apoptosis by modulating mitochondrial membrane potential, ATP synthesis, calcium regulatory proteins, and other associated factors [44]. Additionally, the continuous circulation of Ca^2^⁺ between intracellular and extracellular compartments led to the persistent generation of reactive oxygen species (ROS) [45].

**Figure 4:**
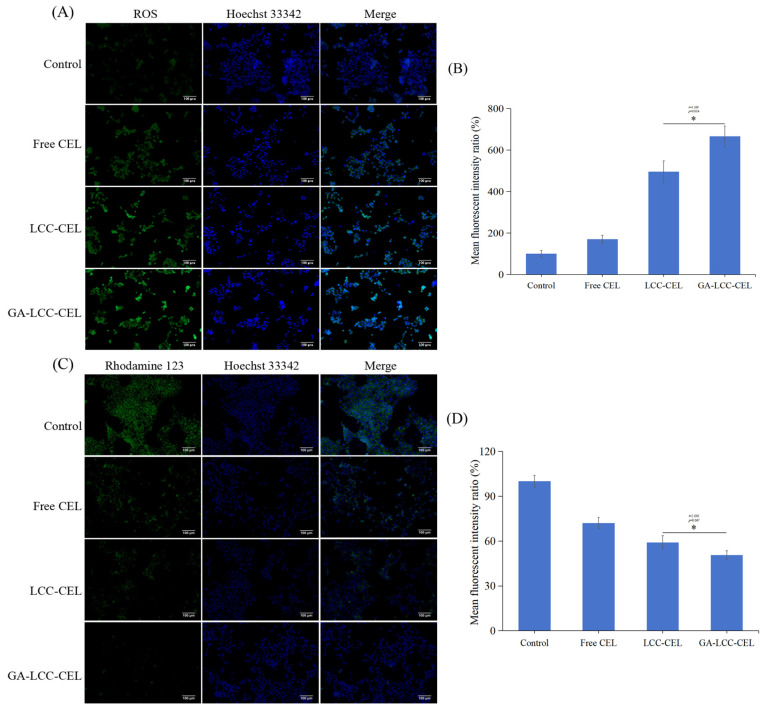
In vitro mitochondrial dysfunction evaluation in various CEL formulations-treated 4T1 cells. (**A**) Intracellular ROS evaluation using DCFH-DA probe in 4T1 cells treated with LCC-CEL, GA-LCC-CEL and free CEL. (**B**) Mean fluorescent intensity of ROS. (**C**) Mitochondrial membrane potential changes of 4T1 cells after different treatments. (**D**) Mean fluorescent intensity of mitochondrial membrane potential. Results are presented as mean ± standard deviation (*n* = 3), * *p* < 0.05.

As shown in Figure 4A,B, intracellular ROS levels in the GA-LCC-CEL group were higher compared to the free CEL and LCC-CEL groups, suggesting that CEL released by GA-LCC-CEL could enhance intracellular ROS production and accumulation. Rhodamine 123 staining was used to further assess mitochondrial membrane potential in 4T1 cells. All CEL groups induced an imbalance in mitochondrial membrane potential, with GA-LCC-CEL exhibiting the most pronounced effect, as shown in Figure 4C,D. These results indicated that GA-LCC-CEL effectively regulated ROS levels and mitochondrial membrane potential imbalance. This effect was likely due to its ability to release Ca^2^⁺, which infiltrated mitochondria and disrupted internal Ca^2^⁺ homeostasis. Consequently, mitochondrial Ca^2^⁺ overload led to the sustained ROS production and modulation of mitochondrial membrane potential, ultimately inducing apoptosis and enhancing the antitumor efficacy of CEL, which was combined with the inherent antitumor activity of GA.

**Figure 5:**
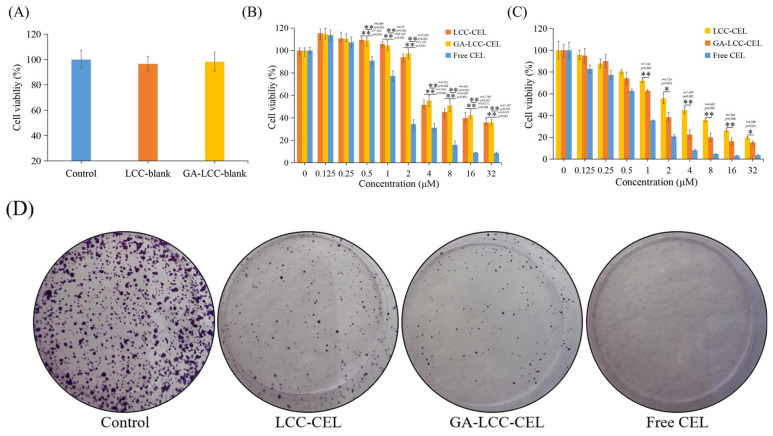

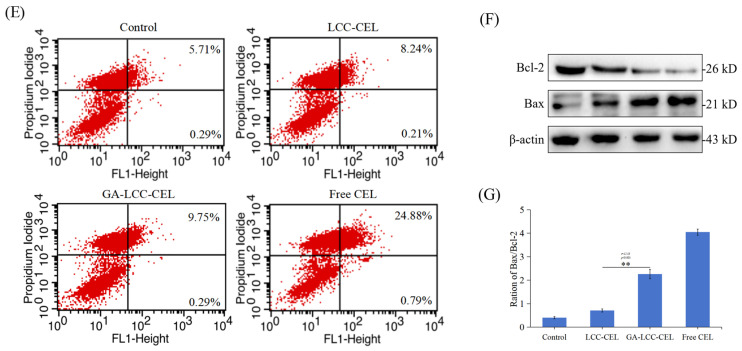
In vitro efficacy evaluation of GA-LCC-CEL nanoparticles. (**A**) In vitro cytotoxicity evaluation of LCC-blank and GA-LCC-blank in MCF-10A cells for 24 h. (**B**) In vitro cytotoxicity evaluation of LCC-CEL, GA-LCC-CEL, and free CEL in MCF-10A cells for 24 h. (**C**) In vitro cytotoxicity evaluation of LCC-CEL, GA-LCC-CEL, and free CEL in 4T1 cells for 24 h. (**D**) Colony formation of 4T1 cells after different treatments. Data are presented as mean ± SD (*n* = 6). * *p* < 0.05, ** *p* < 0.01. (**E**) In vitro cell apoptosis evaluation of LCC-CEL, GA-LCC-CEL, and free CEL in 4T1 cells by flow cytometry. (**F**) Expression levels of pro-apoptotic protein Bax and anti-apoptotic protein Bcl-2 in 4T1 cells incubated with various CEL formulations. (**G**) Quantitative ratio of Bax/Bcl-2 calculated based on band intensity in each group, ** *p* < 0.01.

A nanocarrier with appropriate biocompatibility and non-cytotoxicity is indispensable for its potential application in clinical drug delivery [26]. Using the methyl thiazolyl tetrazolium (MTT) assay, we conducted a preliminary assessment of the cytotoxicity levels of CEL, LCC-blank, and GA-LCC-blank on human normal breast epithelial cells (MCF-10A). As shown in Figure 5A, LCC-blank and GA-LCC-blank demonstrated negligible cytotoxicity on the proliferation of MCF-10A cells at the examined concentrations. Compared to free CEL, the toxicity of GA-LCC-CEL was significantly reduced, indicating that GA modification effectively mitigated CEL-induced damage to normal cells (Figure 5B). As shown in the MTT assay (Figure 5C), all three CEL formulations exerted strong proliferation inhibition effects on 4T1 cells in a concentration-dependent manner. These results were consistent with the cell colony formation test outcomes depicted in Figure 5D. The IC50 values of free CEL, LCC-CEL, and GA-LCC-CEL were calculated to be 0.841 μM (95% CI: 0.775–0.909), 1.109 μM (95% CI: 0.881–1.342), and 2.027 μM (95% CI: 1.415–2.940). Compared to LCC-CEL, GA-LCC-CEL exhibited significantly higher inhibition activity, revealing that cell toxicity was enhanced after GA modification of LCC-CEL. This could be explained by the increased cellular uptake and specific interaction due to the presence of GA.

To assess the impacts of varying concentrations of GA-LCC-CEL on apoptosis in 4T1 cells, the cell populations at four stages—dead cells, late apoptosis, early apoptosis, and live cells—were quantified by flow cytometry following staining with Annexin V-FITC/PI. Comparable outcomes were observed for apoptosis following 24-h exposure to LCC-CEL, GA-LCC-CEL, and free CEL (Figure 5E). The percentages of apoptotic cells in the control group, LCC-CEL, GA-LCC-CEL, and free CEL were 5.32 ± 1.21%, 7.89 ± 1.16%, 10.68 ± 1.36%, and 27.53 ± 2.32%, respectively. Treatment with GA-LCC-CEL significantly increased the rate of apoptosis compared to LCC-CEL, which was consistent with the cytotoxicity results.

Bcl-2 family members, key regulators of cell survival and death, play crucial roles in the regulation of the mitochondrial apoptotic signaling pathway [46]. Bcl-2 and Bax are opposing genes within this family, with Bcl-2 exerting an anti-apoptotic function and Bax playing a pro-apoptotic role [47]. The balance between Bcl-2 and Bax is closely associated with apoptosis. As shown in Figure 5F,G, treatment with LCC-CEL, GA-LCC-CEL, and free CEL resulted in the downregulation of Bcl-2 levels and upregulation of Bax levels compared to the control group. The increase in Bax activity observed in the GA-LCC-CEL group was significantly higher than in the LCC-CEL group, indicating a stronger apoptotic effect induced by GA-LCC-CEL. These findings suggested that the enhanced apoptosis of 4T1 cells following treatment with GA-LCC-CEL could be associated with the activation and regulation of the mitochondrial apoptotic signaling pathway.

### 3.5. In Vitro Cell Migration and Invasion Tests

**Figure 6:**
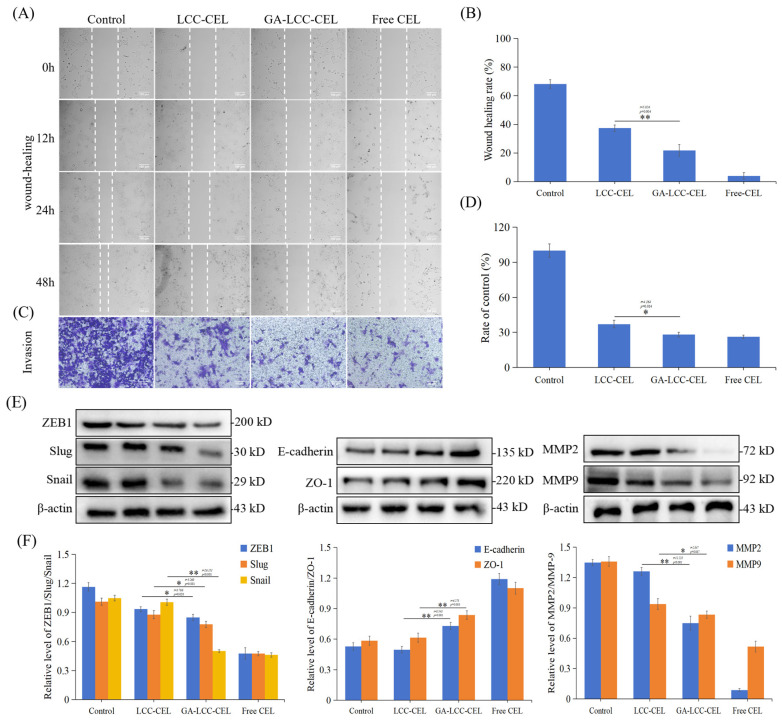
In vitro inhibitory effects of CEL nanoparticles on cell migration and invasion abilities of 4T1 cells. (**A**) Representative images of wound healing assessments of LCC-CEL, GA-LCC-CEL, and free CEL on 4T1 cells. (**B**) Quantified wound healing inhibition rates of various CEL formulations compared to the negative control, ** *p* < 0.01. (**C**) Representative images of invasion assessments of CEL formulations on 4T1 cells. (**D**) Percentage of invading cells quantified and normalized to control group, * *p* < 0.05. (**E**) Expression levels of EMT-related proteins and MMPs in 4T1 cells treated with LCC-CEL, GA-LCC-CEL, and free CEL examined using Western blot analysis. β-actin was used as an internal control. (**F**) Analysis of the band intensity values of the above proteins, * *p* < 0.05, ** *p* < 0.01.

Tumor metastasis involves the dissemination of malignant cells from the primary tumor site to distant organs, encompassing two key mechanisms: tumor cell migration and invasion [48]. Cell migration was assessed using a wound-healing assay (Figure 6A,B). In the control group, the wound scratch demonstrated strong migration ability after 48 h, indicating significant motility in 4T1 cells. Notably, free CEL, which was used as a positive control, showed the most pronounced inhibition of cell motility, with only a 3.94% wound healing rate. The wound healing rates following treatment with LCC-CEL and GA-LCC-CEL were 37.31% and 21.78%, respectively. These findings suggested that GA-LCC-CEL exhibited a significantly enhanced inhibitory effect on tumor cell migration compared to LCC-CEL. This enhanced inhibitory effect of GA-LCC-CEL could be attributed to the modification of the GA-mediated endocytic pathway on the liposome surface, leading to increased uptake by 4T1 cells.

GA-LCC-CEL exhibited a similar inhibitory effect in transwell invasion assays. As shown in Figure 6C,D, the inhibitory effect of GA-LCC-CEL was equally potent compared to LCC-CEL in 4T1 cells. Specifically, only about 28.05% of the cells in the GA-LCC-CEL group successfully crossed the Matrigel membrane, which was significantly lower than the 37.09% observed in the LCC-CEL group.

The process of epithelial–mesenchymal transition (EMT) is crucial for normal embryonic development and acts as a primary trigger for tumor invasion and metastasis. During EMT, significant alterations occur in the cytoskeleton and cell signaling pathways [49,50]. A key characteristic of EMT is the loss of epithelial integrity, marked by a reduction in adhesive junctions that maintain epithelial-cell contact [51]. This transition involves the upregulation of specific transcription repressors, such as Snail, Slug, and ZEB1, which suppress E-cadherin expression. Additionally, EMT is associated with the disruption of the essential tight junction protein ZO-1 and the enzymatic degradation of extracellular matrix components by matrix metalloproteinases (MMPs), including MMP2 and MMP9. These changes lead to cellular dissociation and increased motility [52,53].

To further investigate the anti-migration mechanism of GA-LCC-CEL, we performed Western blot analysis to assess the protein expression levels of EMT-related markers. As depicted in Figure 6E,F, GA-LCC-CEL significantly upregulated E-cadherin and ZO-1 proteins while downregulating the Snail, Slug, and ZEB1 proteins in 4T1 cells. Compared to the control group, GA-LCC-CEL led to 38.24% and 43.34% increases in the E-cadherin and ZO-1 expression levels, along with 27.15%, 23.25%, and 52.19% decreases in the ZEB1, Slug, and Snail expression levels, respectively. LCC-CEL slightly regulated both E-cadherin and ZO-1 and slightly decreased the expression levels of Snail, Slug, and ZEB1. Furthermore, GA-LCC-CEL effectively inhibited the expression of the MMP2 and MMP9 proteins. These findings collectively indicated that GA-LCC-CEL could inhibit cell migration and invasion by modulating the EMT signaling pathway in 4T1 cells.

### 3.6. Inhibitory Effect on the Tumor Spheroids

**Figure 7:**
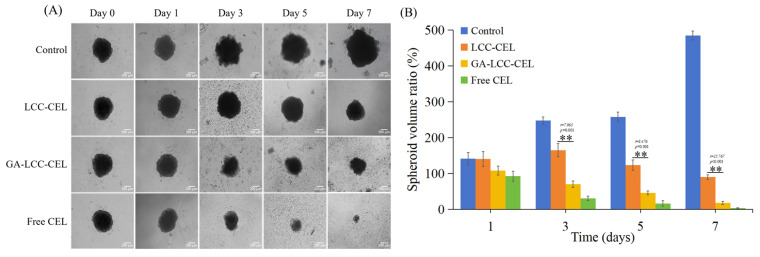
In vitro inhibitory effect on 4T1 tumor spheroids. (**A**) Morphological changes in 4T1 tumor spheroids during 7 days of treatment with LCC-CEL, GA-LCC-CEL, and free CEL. (**B**) Quantified size changes of 4T1 tumor spheroids after treatment with different formulations. Data are presented as mean ± SD (*n* = 3), ** *p* < 0.01.

The multi-cellular tumor spheroid (MCTS) is a three-dimensional tumor cell culture model that mimics solid tumors in vivo and simulates the pathophysiological characteristics of human tumor tissue through cell-to-cell, cell-to-matrix, and three-dimensional network interactions [54]. This widely used model has been instrumental in antitumor drug research [55]. Therefore, this study employed the MCTS model to investigate the inhibitory impacts of various CEL modifications. As anticipated (Figure 7A,B), GA-LCC-CEL exhibited greater efficacy in reducing tumor spheroid volume compared to LCC-CEL, indicating its superior inhibitory potential. These results suggested that the inhibitory effect of GA-LCC-CEL could be attributed to GA modification, effectively suppressing cell proliferation in both 2D monolayers and 3D spheroids. These findings provided direct evidence that GA-LCC-CEL nanoparticles could deeply penetrate tumor tissues while maintaining a sufficient drug concentration for effective chemotherapy.

## 4. Conclusions

In contrast to previous studies on the antitumor effects of conventional liposomes loaded with camptothecin (CEL) [56,57,58], in this study, we have utilized calcium carbonate nanoparticles as effective drug carriers and prepared liposomes using GA as the lipid material to encapsulate calcium carbonate nano-ions to make a carrier system for the targeted delivery of CEL, known as GA-LCC-CEL; this possessed significant characteristics, such as an appropriate size and charge, higher stability and sustained release properties. These features enable the targeted delivery of CEL to 4T1 cells. Additionally, the nanoparticles modified with GA can mitigate the damage to normal cells induced by CEL. The subsequent disintegration of CCNP-CEL leads to the rapid release of CEL and Ca^2+^. The excessive influx of Ca^2+^ into mitochondria results in mitochondrial dysfunction and apoptosis induction. As a result of the inherent antitumor effect of GA and its mediated endocytosis, GA-LCC-CEL significantly enhances intracellular uptake efficiency, cytotoxicity, and pro-apoptotic activity compared to LCC-CEL. Importantly, GA-LCC-CEL exhibits significant inhibitory effects on tumor cell invasion and migration by regulating the expression levels of EMT-related proteins. These findings suggest that GA-LCC-CE investigated in this study facilitates the rapid release of CEL, GA, and Ca^2+^ within tumor cells. These components synergistically enhance the antitumor effect, indicating the potential of GA-LCC-CE as a targeted drug delivery strategy to improve therapeutic efficacy in metastatic breast cancer. This may be attributed to the amphiphilic structure of GA, which, similar to surfactants, can interact with phospholipids on the cell membrane, thereby altering the fluidity of lipids and enhancing the permeability of the cell membrane and the transmembrane ability of drugs; this facilitates the entry of CEL into cells. In addition, the inherent antitumor activity and tumor-targeting capability of GA enable the released CEL and GA from GA-LCC-CEL to exert synergistic antitumor effects, thereby improving therapeutic efficacy.

## Data Availability

Data are available upon request from the first author (Wei Zhang, zhv1110@qmu.edu.cn).
